# Melatonin prevents acute kidney injury in severely burned rats via the activation of SIRT1

**DOI:** 10.1038/srep32199

**Published:** 2016-09-07

**Authors:** Xiao-Zhi Bai, Ting He, Jian-Xin Gao, Yang Liu, Jia-Qi Liu, Shi-Chao Han, Yan Li, Ji-Hong Shi, Jun-Tao Han, Ke Tao, Song-Tao Xie, Hong-Tao Wang, Da-Hai Hu

**Affiliations:** 1 Department of Burns and Cutaneous Surgery, Xijing Hospital, The Fourth Military Medical University, 127 Changle West Road, Xi’an, Shaanxi 710032, China

## Abstract

Acute kidney injury (AKI) is a common complication after severe burns. Melatonin has been reported to protect against multiple organ injuries by increasing the expression of SIRT1, a silent information regulator that regulates stress responses, inflammation, cellular senescence and apoptosis. This study aimed to investigate the protective effects of melatonin on renal tissues of burned rats and the role of SIRT1 involving the effects. Rat severely burned model was established, with or without the administration of melatonin and SIRT1 inhibitor. The renal function and histological manifestations were determined to evaluate the severity of kidney injury. The levels of acetylated-p53 (Ac-p53), acetylated-p65 (Ac-p65), NF-κB, acetylated-forkhead box O1 (Ac-FoxO1), Bcl-2 and Bax were analyzed to study the underlying mechanisms. Our results suggested that severe burns could induce acute kidney injury, which could be partially reversed by melatonin. Melatonin attenuated oxidative stress, inflammation and apoptosis accompanied by the increased expression of SIRT1. The protective effects of melatonin were abrogated by the inhibition of SIRT1. In conclusion, we demonstrate that melatonin improves severe burn-induced AKI via the activation of SIRT1 signaling.

A severe burn injury often leads to systemic inflammatory response syndrome (SIRS), sepsis, acute kidney injury (AKI) and multiple organ dysfunction syndrome (MODS). AKI is a leading complication in patients with extensive deep burns in which burned area exceeds 20% of the total body surface area (TBSA)^1^,^2^. However, the pathophysiologic mechanism underlying burn-induced AKI remains incompletely elucidated. An increasing number of evidences have shown that the inflammatory response and oxidative stress play a key role in the development of AKI. The pro-inflammatory cytokines such as tumor necrosis factor-α (TNF-α), interleukin -1β (IL-1β) and cell adhesion molecules are overexpressed after severe burns, leading to uncontrolled inflammatory response and organ injury. The excessive production of reactive oxygen species (ROS) can lead to cell damage and finally result in organ failure. Furthermore, ROS overproduction leads to mitochondrial dysfunction and adenosine triphosphate (ATP) depletion^3^, triggering cytochrome c to leak from the mitochondria into the cytoplasm and ultimately causing cell apoptosis^4^.

Melatonin (N-acetyl-5-methoxytryptamine), a circadian hormone mainly secreted by the pineal gland, is a derivative of tryptophan and predominantly secreted during night^5^. Melatonin or its derivative has been reported to exert various biological activities like anti-oxidative^6^,^7^,^8^,^9^, anti-inflammatory^10^,^11^,^12^,^13^ and anti-apoptotic^14^,^15^,^16^ effects. Recently, melatonin is widely used to protect organs against endogenous damage due to its lack of toxicity. Melatonin has also been reported to protect kidney against injury caused by ROS^17^,^18^. Others have reported that melatonin can act as antioxidant to protect against organ damage induced by severe burns^19^,^20^,^21^,^22^. However, the specific mechanism by which melatonin protects kidney against severe burn-induced injury is still unclear.

SIRT1 plays a role in transcriptional and post-transcriptional regulation of gene expression through the deacetylation of histone and non-histone proteins^23^. Recent data suggest that SIRT1 targets p53^24^, FoxO1^25^, and NF-κB^26^ for deacetylation and thus regulates stress responses, inflammation, cellular senescence and apoptosis^27^. Interestingly, melatonin has been reported to promote the expression of SIRT1 and protect against nerve injury and ischemia-reperfusion injury in myocardium^28^,^29^,^30^. Melatonin shows protective effects on organ dysfunction, however, the role of SIRT1 and SIRT1 related signaling pathway responsible for the former effects is still unclear. This study focused on the point that whether melatonin is protective in acute kidney injury (AKI) induced by severe burns and whether the protective effects are associated with the activation of SIRT1.

## Results

### Melatonin ameliorates renal damage in rats after severe burn

**Figure 1:**
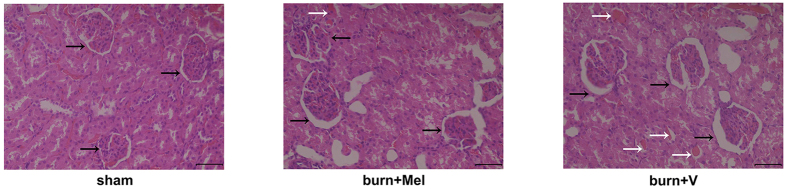
Histopathological findings of severe burn induced kidney injury. Twenty-four hours after the burn injury, rats were sacrificed and kidneys were harvested and subjected H&E staining. The black arrows direct to Bowman’s capsule. The percentages of necrotic glomeruli were increased in burn group than those in burn + melatonin group. The percentages of tubular containing casts were increased in burn group compared to those in normal group and burn + melatonin group. Statistical analysis was performed by Mann Whitney U test (*p* < 0.05). n = 8 in each group. Scale bars in right lower corner represent 100 μm. Black arrow: Bowman’s capsule; White arrow: Cast. Sham = sham control; burn + v = burn + vehicle group; burn + mel = burn + melatonin group.

Twenty-four hours after burns, the rats were sacrificed and the H&E staining was used to detect the pathological changes of kidney. In the control group, the morphology of glomerulus was normal with no cast formation in the tubular. The percentages of necrotic glomeruli were increased in burn group than those in burn + melatonin group. The percentages of tubular containing casts were increased in burn group compared to those in sham group and burn + melatonin group (*p* < 0.05) (Fig. 1, Figs S1 and S3).

### Melatonin improves the kidney function and protects against oxidative damage after severe burn

**Figure 2:**
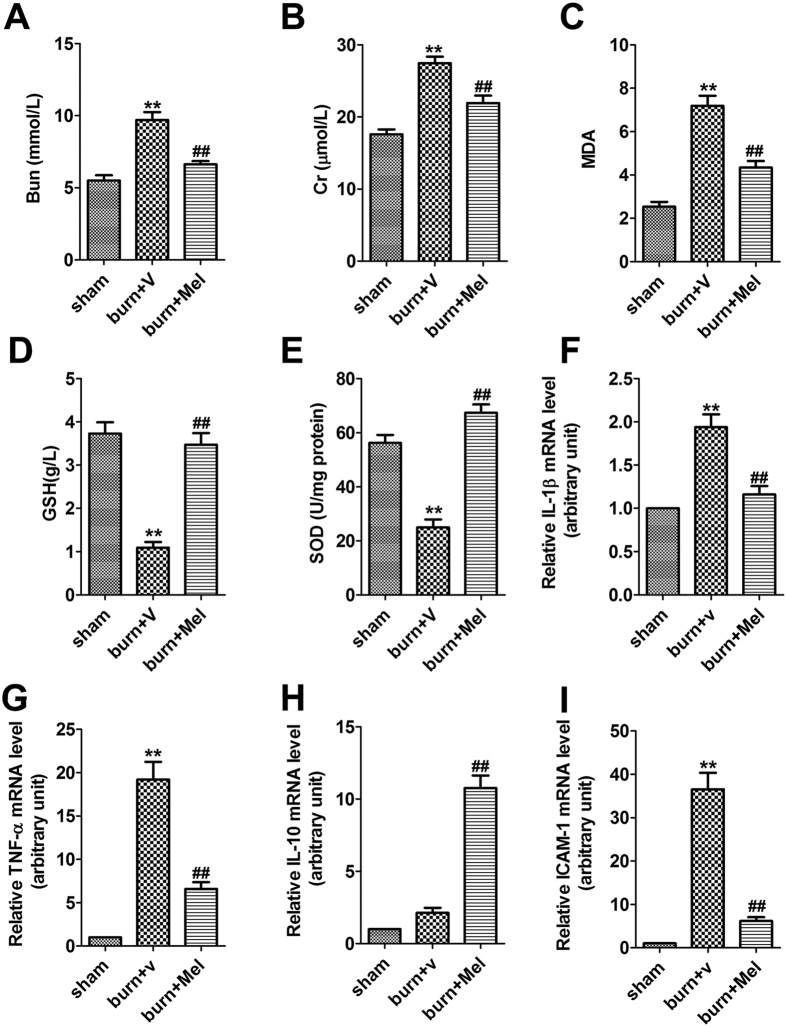
Melatonin improves kidney function and decreases the level of oxidative related molecules and pro-inflammation cytokines. **(A**,**B**) The serum levels of Cr and BUN in burned rats, as determined using a clinical chemistry analyzer. (**C**–**E**) Melatonin decreased the expression of MDA but increased the levels of GSH and SOD in renal tissues. (**F**–**I**) The mRNA levels of TNF-α, IL-1β, IL-10, and ICAM-1 in renal tissues. mRNA was obtained from renal tissues of different groups following 24 h of burn injury. n = 8 in each group. Ct values were normalized to the expression of the GAPDH gene. Differences were calculated with the 2^−∆∆Ct^ method and data are expressed as the percentage relative to the values obtained for the sham group. The expressions of TNF-α, IL-1β, and ICAM-1 in burn group were increased while the expression of IL-10 did not change significantly. The expression of TNF-α, IL-1β, and ICAM-1 were significantly decreased after the administration of melatonin. The expression of IL-10 increased in the burn + melatonin group. n = 8 in each group. **p < 0.05, compared to the value of sham group; ^##^*p* < 0.05, compared to the value of burn + vehicle group.

The Cr and BUN levels in serum were significantly increased after severe burns (more than 1.5-fold). However, melatonin administration significantly decreased the levels of Cr and BUN in the serum of burned rats (Fig. 2A,B). We investigated the expression of MDA, a biomarker of the level of oxidative stress, in the kidney tissue. The results showed that the expression of MDA in burn + vehicle group was increased by 2.8-fold, while melatonin admistration significantly decreased the burn-induced MDA expression (Fig. 2C). In burn + vehicle group, burn injury resulted in a significant reduction of SOD and GSH expression in renal tissues, however, the expression of SOD and GSH in burn + Mel group was similar to that in the sham group (Fig. 2D,E).

### Melatonin inhibits the burn-induced expression of pro-inflammatory cytokines in renal tissues

Twenty-four hours after burns, the mRNA levels of TNF-α, IL-1β, IL-10 and ICAM-1 in renal tissue were detected by RT-PCR. In the burn + vehicle group, the expression of TNF-α, IL-1β, and ICAM-1 were increased while the expression of IL-10 did not change significantly (Fig. 2F–I). The expression of TNF-α, IL-1β, and ICAM-1 were significantly decreased after the administration of melatonin. Notably, the expression of IL-10 was increased in the burn + melatonin group (Fig. 2H).

### Melatonin suppresses cell apoptosis in renal tissue after severe burns

**Figure 3:**
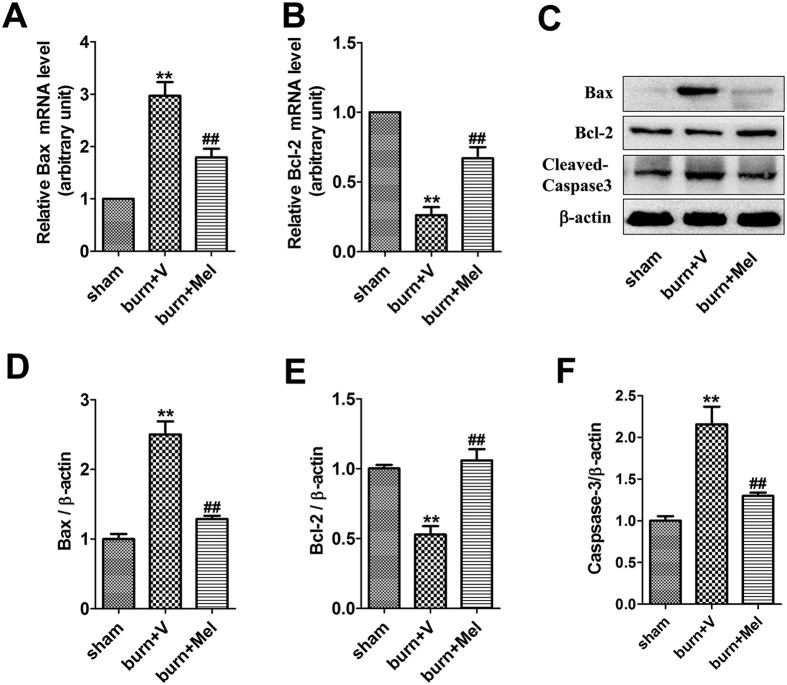
Melatonin suppressed the apoptosis of cells in renal tissue after severe burn. **(A**,**B**) The mRNA level of Bax in burn + melatonin group decreased compared to burn group. However, that of Bcl-2 increased in burn + melatonin group. (**C**–**F**) The protein levels of Caspase-3 increased significantly after severe burns, which could be decreased by the administration of melatonin. The proteins level of Bcl-2 and Bax decreased in burn group and increased in burn + melatonin group. n = 8 in each group. **p < 0.05, compared to the value at sham group; ^##^*p* < 0.05, compared to the value at burn + vehicle group.

We next evaluated cell apoptosis in renal tissue after severe burns. The protein levels of anti-apoptotic Bcl-2 and pro-apoptotic Bax were detected through Western Blot and the related mRNA levels were detected by RT-PCR. Melatonin increased the expression of Bcl-2 compared to burn + vehicle group, while the expression of Bax was increased after severe burns but decreased after the administration of melatonin (Fig. 3A–E). The protein level of caspase-3 was increased by 2-fold after severe burns, which was suppressed by the administration of melatonin. As shown in Fig. S5, the number of apoptotic renal tubular epithelial cells rose significantly after burns. However, compared with the burn group, the apoptosis of cells were significantly decreased after the administration of melatonin. The apoptotic index of burn group was 2 to 3 fold than the burn + melatonin group (p < 0.05).

### Melatonin promotes the expression of SIRT1 in renal tissues and increases the deacetylation of FoxO1, p65 and p53

**Figure 4:**
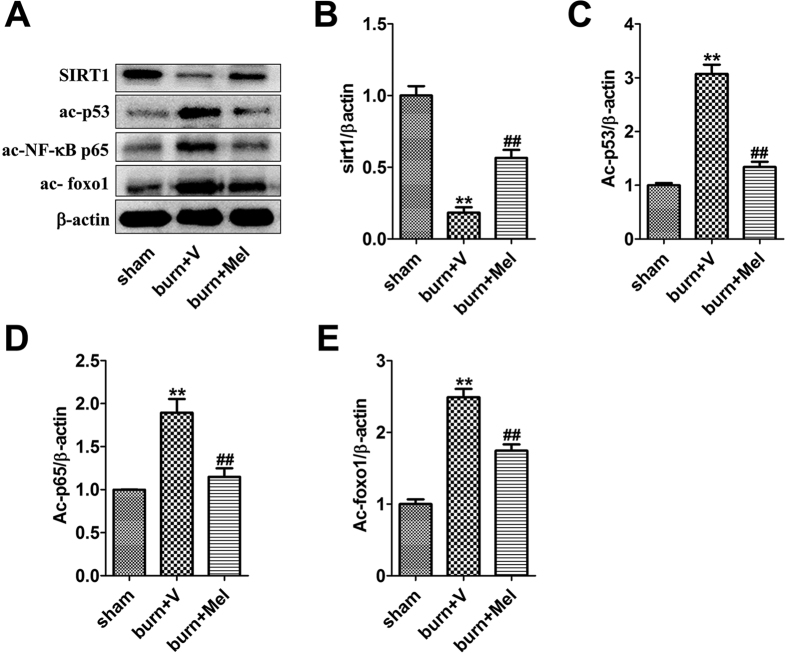
Melatonin promotes the expression of SIRT1, but decreases the levels of ac-FoxO1, ac-NF-κB and ac-p53 in renal tissues. The western blot showed that the expression of SIRT1 decreased significantly in burn group. The acetylation of FoxO1, NF-κB and p53 was suppressed in the meantime. n = 8 in each group. **p < 0.05, compared to the value at sham group; ^##^*p* < 0.05, compared to the value at burn + vehicle group.

Notably, the expression of SIRT1 was decreased and the acetylation of FoxO1, p65 and p53 was increased in renal tissues of burn + vehicle group (Fig. 4). The administration of melatonin resulted in up-regulation of SIRT1 and increased deacetylation of FoxO1, p65 and p53 (Fig. 4).

### Melatonin could protect renal tissues through the activation of SIRT1

**Figure 5:**
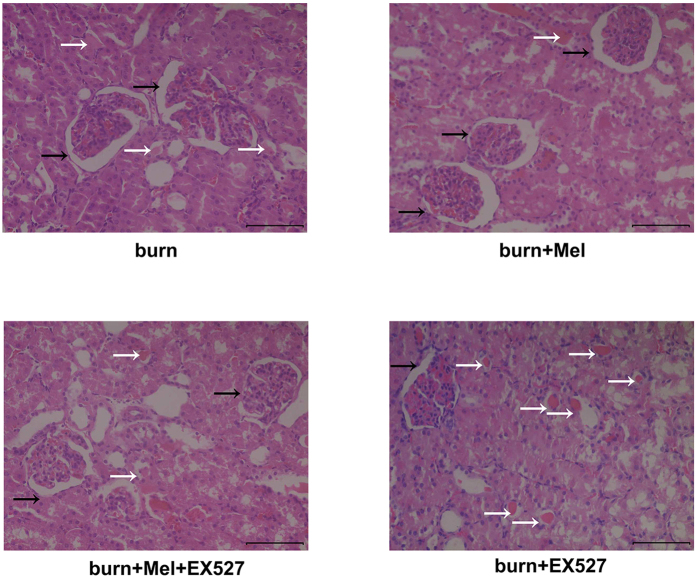
EX527 abrogates the protective effects of melatonin against burn-induced renal injuries. The HE staining showed that there were more necrotic glomeruli in burn + melatonin + EX527 group than in burn + melatonin group. The percentages of tubular containing casts were increased in burn + melatonin + EX527 group compared to those in burn + EX527 group. Black arrow: Bowman’s capsule; White arrow: cast. Statistical analysis was performed by Mann Whitney U test (*p* < 0.05). There is no significant difference between burn + vehicle group and burn + EX527 group. n = 8 in each group. Scale bars in the right lower corner represent 100 μm.

**Figure 6:**
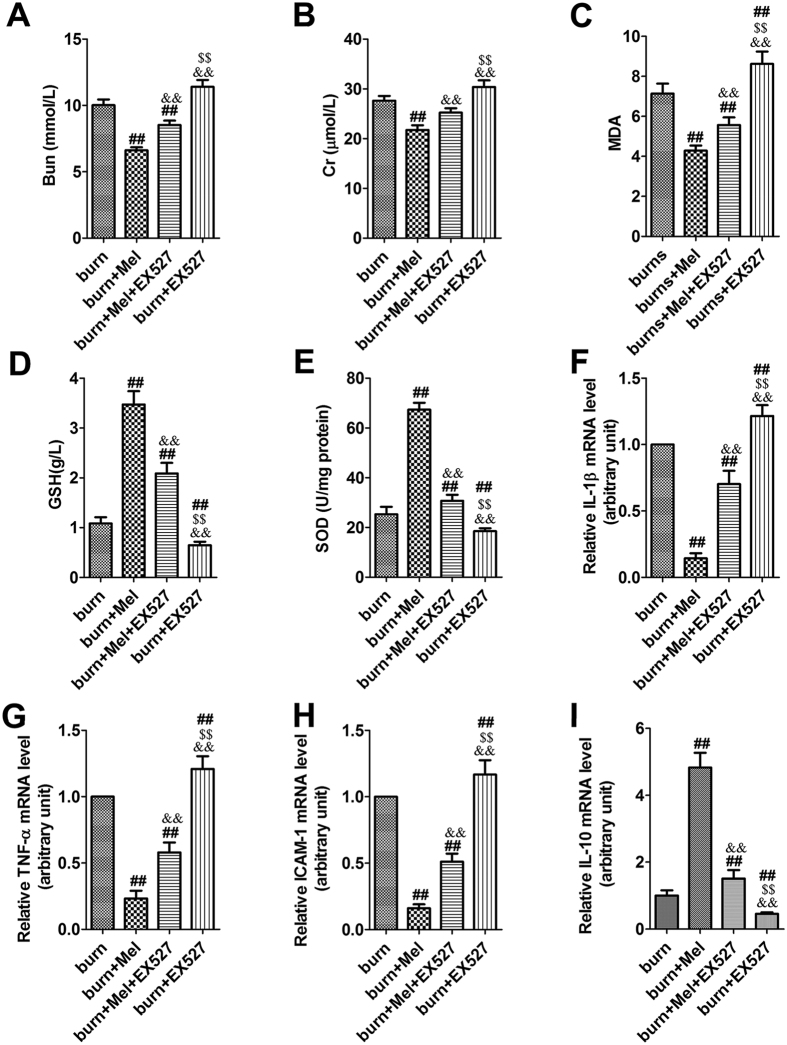
Inhibition of SIRT1 suppresses the effects of melanin on the renal tissues of burned rats. (**A**,**B**) The creatinine and urea nitrogen level in serum was significantly higher in burn group compared to burn + melatonin group and sham group. (**C**) The expression level of MDA was increased in burn group and decreased in burn + melatonin group. (**D**,**E**) The accumulation of SOD and GSH increased to a level similar to the control group compared with burn group. (**F**–**I**) The expression of TNF-α, IL-1β, and ICAM-1in burn group were increased while the expression of IL-10 did not change significantly. The expression of TNF-α, IL-1β, and ICAM-1 were significantly decreased after the administration of melatonin. The expression of IL-10 increased in the burn + melatonin group. n = 8 in each group. ^##^*P* < 0.05 compared to the value at the burn group, ^&&^*P* < 0.05 compared to the value at the Melatonin + burn group, ^$$^*P* < 0.05 compared to the value at the Melatonin + EX527 + burn group.

To further investigate whether SIRT1 is responsible for the protection effects of melatonin, EX527, the specific antagonist of SIRT1, was administrated to rats after severe burns. Dramatically, the administration of EX527 decreased the protective effects of melatonin on renal tissues against severe burns. As is shown in Fig. 5, compared to burn + melatonin group, renal tissues in burn + melatonin + EX527 group showed obvious contraction of glomerulus and dilatation of Bowman’s capsule, which means there were more necrotic glomeruli in burn + melatonin +EX527 group than in burn + melatonin group. The percentages of tubular containing casts were increased in burn + melatonin + EX527 group compared to those in burn + EX527 group (p < 0.05) (Fig. 5, Figs S2 and S4). The Cr and BUN level in serum increased significantly in burn + melatonin + EX527 group compared to burn + melatonin group (Fig. 6A,B).

### Melatonin could attenuated the inflammatory response, oxidative stress and cell apoptosis through the activation of SIRT1

**Figure 7:**
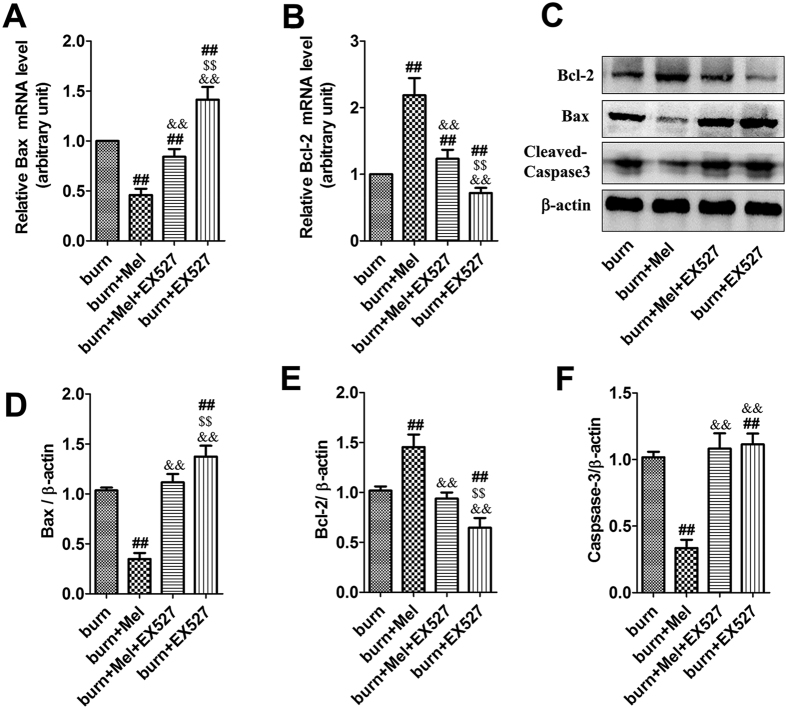
Inhibition of SIRT1 suppresses the anti-apoptotic effects of melatonin on the renal tissues of burned rats. (**A**,**B**) The mRNA levels of Bax and Bcl-2 in renal tissue. Melatonin could decrease the expression of Bax while administration of EX527 increased the expression of Bax, while the administration of melatonin could upregulate the expression of Bcl-2, which could be reversed by EX527. (**C**–**F**) The protein levels of Bax, Bcl-2 and cleaved-caspase-3 in renal tissues. Data are expressed as the percentage relative to the values obtained for the burn group. n = 8 in each group. ^##^*P* < 0.05 compared to the value at the burn group, ^&&^*P* < 0.05 compared to the value at the Melatonin + burn group, ^$$^*P* < 0.05 compared to the value at the Melatonin + EX527 + burn group.

**Figure 8:**
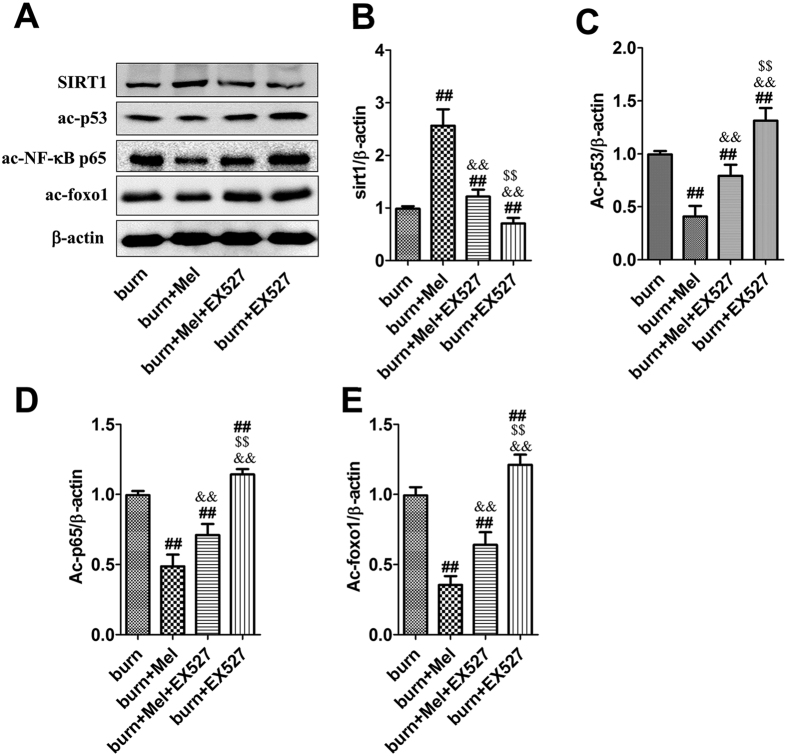
Inhibition of SIRT1 decreases the deacetylation of NF-κB, FoxO1 and p53 in renal tissues. The protein levels of ac-NF-κB p65, ac-FoxO1 and ac-p53 in renal tissues were measured by western blot, which showed that the expression of SIRT1 increased in burn + melatonin group while decreased after the administration of melatonin. The acetylation of NF-κB, FoxO1 and p53 showed an opposite pattern compared to that of SIRT1. ^##^*P* < 0.05 compared to the value at the burn group, n = 8 in each group. ^&&^*P* < 0.05 compared to the value at the Melatonin + burn group, ^$$^*P* < 0.05 compared to the value at the Melatonin + EX527 + burn group.

MDA, one of the advanced lipoxidation end products (ALEs), was decreased in renal tissues of the burn + melatonin group compared to the burn group. However, the administration EX527 significantly increased burn-induced production of MDA (Fig. 6C). SOD and GSH, two key endogenous antioxidants, were increased in burn + melatonin group compared with burn group (Fig. 6D,E). The administration of EX527 decreased the expression levels of SOD and GSH significantly. IL-1β and TNF-α, both of which were important inflammatory cytokines, were decreased after the administration of melatonin and increased when treated with the combination of melatonin and EX527 (Fig. 6F,G). ICAM-1, an adhesion molecule, was decreased by melatonin, while the administration of EX527 significantly up-regulated its expression. When EX527 was used alone after burn injury, the expression of ICAM was also increased significantly (Fig. 6H). The anti-inflammatory cytokine IL-10 showed an opposite pattern compared to that of IL-1β and TNF-α (Fig. 6I). The protein levels of cleaved-caspase-3 and BAX, two apoptotic biomarkers, showed an identical pattern compared to the mRNA level of TNF-α (Fig. 7). The acetylation of NF-κB, FoxO1 and p53 in the presence or absence of EX527 was determined to test our hypothesis that SIRT1 deacetylates related proteins and then exerts cytoprotective effects. It was showed that the expression of SIRT1 in renal tissues was increased by 2.6-fold when the burned rats were treated with melatonin, but decreased significantly when treated with EX527, as well as the combination of melatonin and EX527. Importantly, the acetylation of NF-κB, FoxO1 and p53 showed an opposite pattern compared to that of SIRT1 (Fig. 8).

## Discussion

Multiple organ dysfunction syndrome (MODS) induced by severe burns are critical conditions with an incidence of nearly 28%^31^. The circulatory blood volume decreases rapidly after severe burns, which leads to microcirculation disturbance, ischemic and anoxia changes of multiple organs. Kidney is one of the most frequently involved organs in that circumstance. It is reported that patients suffered from burns exceeded 20% of body surface tend to develop AKI, even acute kidney failure, which is one of the most important causes of MODS and death^1^,^2^.

AKI is typically presented as a decrease of renal perfusion pressure and the induction of acidosis, and then leading to glomerulus contraction and renal tubular necrosis. In the rat burn model of our study, the H&E staining showed apparent dilatation of Bowman’s capsule in renal tissue 24 hours after severe burns, accompanied by the formation of casts in renal tubular. The expansion of Bowman’s capsule and the apparent of casts are important manifestations of kidney injury^32^,^33^. Additionally, both Cr and BUN were increased after severe burns, indicating that the glomerular filtration rate (GFR) decreased to less than 60% compared to normal kidney. However, the administration of melatonin improved the morphological manifestation of renal tissues and decreased the accumulation of Cr and BUN in the blood. The findings indicate that melatonin did improve the renal function after severe burns.

Oxidative stress plays a critical role in the development of AKI^34^,^35^,^36^. ROS are the major factors and the last transmitters downstream of several cytokines leading to kidney injury^37^, which lead to membranous lipid peroxidation, protein oxidation, DNA damage, and the exhaustion of endogenous antioxidant enzymes and finally result in cell death and organ damage. This procedure is one of the important mechanisms of AKI in early stage of burns. MDA is one of the important ALEs usually used as a scale to determine the severity of oxidative stress. SOD and GSH act as endogenous antioxidants and prevent the attacks of activated oxygen by targets the superoxide radicals. In the present research, MDA was increased significantly while SOD and GSH decreased dramatically during severe burns. Melatonin decreased the expression level of MDA and enhanced the expression of SOD and GSH. The results imply that melatonin might improve the renal function through the inhibition of oxidative stress.

In addition, inflammation also plays a role in the progression of renal injuries induced by severe burns. Pro-inflammatory cytokines such as TNF-α, IL-1β and IL-6, and adhesion molecule ICAM-1 can trigger strong inflammatory reactions in response to injurious stimuli^38^. In the rat severe burn model, the mRNA level of TNF-α, IL-1β and ICAM-1 in renal tissue was increased significantly. On the contrary, the level of IL-10, an anti-inflammatory cytokine^39^,^40^, was significantly suppressed in the kidney of severely burned rats and recovered after the administration of melatonin. Based on the results we deduced that melatonin could attenuate the inflammatory response through inhibiting the expression of pro-inflammatory cytokines and up-regulating the expression of anti-inflammatory cytokines.

Cell death, including apoptosis and necrosis, is a common response in renal tissues when confronting injuries such as burns, ischemia, radiation, trauma, and toxicants^41^,^42^,^43^,^44^. Mariano *et al*.^43^ have demonstrated that pro-apoptotic mediators in the circulatory system contribute to renal functional alterations after burns. Multiple cytokines are involved in the regulation of apoptosis, including pro-apoptotic factors like Bax/Bak/Fas and anti-apoptotic factors like Bcl-2/Bcl-xl. In severe burned rats, the expression of Bax was increased significantly while that of Bcl-2 decreased dramatically. The administration of melatonin decreased the expression of pro-apoptotic factor Bax and promoted the expression of anti-apoptotic factor Bcl-2. Meanwhile, the level of cleaved-caspase-3 was increased significantly after burns but was decreased by melatonin. TUNEL staining was used to further illustrate cell apoptosis induced by severe burns. As shown in Fig. S5, the apoptotic tubular epithelial cells were significantly increased after burns which were coincidence with other references focused on renal injury after burns^45^. The administration of melatonin could improve the situation. In summary, melatonin might protect against AKI by inhibiting injurious stimuli-induced cell apoptosis.

Our former research discovered that the activation of SIRT1 could improve burn-induced lung injury^46^. Considering that SIRT1 is an important nicotinamide adenine dinucleotide (NAD^+^)-dependent deacetylase which regulates stress responses, inflammation, apoptosis and cellular senescence, and plays an important role in the development of several renal diseases^47^,^48^,^49^,^50^,^51^,^52^, we investigated its expression in rat renal tissue after severe burns. It was showed that the level of SIRT1 was decreased after burns and recovered after the administration of melatonin. EX527, the specific inhibitor of SIRT1, effectively abrogated the protective effects of melatonin on renal function, leading to our speculation that SIRT1 may play a role in the protective effects of melatonin.

SIRT1 plays an important role in the development of several renal diseases^47^,^48^,^49^,^50^,^51^,^52^, and its activation reduces the occurrence of AKI induced by drugs, toxicants and ischemic-reperfusion injury. In mouse proximal tubular epithelial cell model, the administration of cis-platinum leads to the inhibition of SIRT1 as well as ac-p53, PUMA-α, Bax and cleaved-caspase-3, while resveratrol can reverse the effects^47^. However, the knockout of SIRT1 blocks the protection effect of resveratrol. Hasegawa *et al*. have reported that SIRT1 expressed in the proximal tubules can decrease the level of ROS in local through increasing the expression of catalase and finally improve AKI induced by ischemic reperfusion injury^50^. However, the protection mechanism of SIRT1 in severe burn induced kidney injury is still unclear.

It has been reported that SIRT1 can combine with FoxO1 and participate in the deacetylation of FoxO1^53^. The deacetyation of FoxO1 suppresses the expression of apoptosis related molecules and increases the synthesis of antioxidant such as SOD, which is a powerful oxidants protecting cells against injury^25^,^54^. SIRT1 has also been reported to deacetylate NF-κB subunit p65 to decrease the transcription of NF-κB^26^, which participates in apoptosis, aging, inflammation and inherent immunity, and then reduce cell apoptosis and inflammation. Additionally, p53, one of the substrates of SIRT1, could combined with SIRT1, resulting in the deacetylation of lysine residues of C terminal domains, leading to decreased capability of p53 to activate downstream genes, and finally reducing the cell apoptosis^24^,^55^. In our study, the acetylation of FoxO1, NF-κB, p65 and p53 was increased in severe burn-induced AKI, but decreased when administrated with melatonin, accompanied by increased expression of anti-apoptotic protein Bcl-2 and decreased expression of pro-apoptotic protein Bax. However, these effects were reversed by EX527, the specific inhibitor of SIRT1, which indicates that the activation of SIRT1 is the key factor that regulates the deacetyation of FoxO1, NF-κB, p65 and p53, and subsequently affect the inflammatory response, oxidative stress and cell apoptosis in the involved tissues.

**Figure 9:**
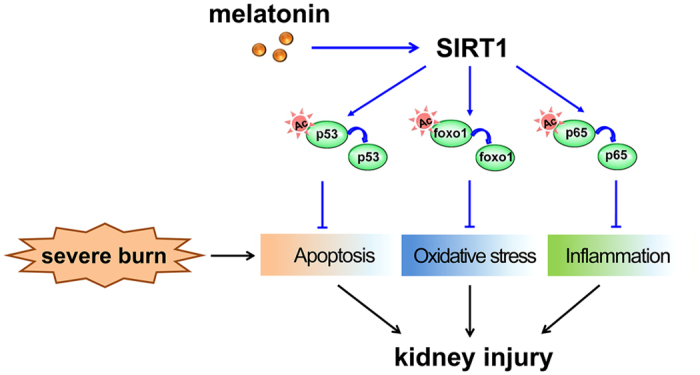
Proposed mechanisms underlying the therapeutic effects of melatonin on severe burn-induced AKI.

In the present study, we first demonstrated the protective effects of melatonin to kidney in a rat severe burn model. The results showed that melatonin could protect against AKI through the activation of SIRT1. The latter could alter the acetylation of FoxO1, p53 and NF-κB, and decrease the ROS accumulation as well as inhibit inflammatory response and tubular epithelial cell apoptosis, which finally ameliorates AKI induced by severe burns (Fig. 9). Our study provides evidence supporting the therapeutic potential of melatonin to improve organ function after severe burns.

## Materials and Methods

### Animals

Healthy adult male Sprague-Dawley (SD) rats weighing 200~250 g were included in this study. Animals were provided by Experimental Animal Center of The Fourth Military Medical University. Rats were fed *ad libitum* a standard diet and water throughout the study. All animals were housed separately and kept under standard conditions at room temperature (22~24 °C) under a 12:12 h light/dark cycle. All of the protocols were carried out in accordance with the approved guidelines in the ethical permit approved by the Ethics Committee of Xijing Hospital, affiliated with the Fourth Military Medical University (XJYYLL-2015206).

### Severe burn model

Rats were randomly divided into five group (n = 8). After a SD rat was anaesthetized with sodium pentobarbital (30 mg/kg, intraperitoneal injection), the shaved back of the rat was immersed into 98 °C hot water for 15 s, generating a full-thickness dermal burn model with 40% TBSA^56^. The sham group was exposed to 25 °C water for 15 s after anesthesia. Liquid resuscitation with lactated Ringer solution (LRS) at 50 mg/kg with or without drugs was injected intraperitoneally immediately after burns and 6 h afterwards. All rats were housed in individual cages and given 0.25 mg/kg buprenorphine by subcutaneous injection immediately and every 12 h post burn for analgesia.

### Reagents

Melatonin (Mel), EX527, rat GSH and MDA kits were purchased from Sigma-Aldrich. BUN and Cr kit was purchased from Beyotime (Shanghai, China). Antibodies against SIRT1, acetylated-p53 (Ac-p53), acetylated-NF-κB (p65), (Ac-NF-κB), acetylated-forkhead box O1 (Ac-FoxO1), Bcl-2, Bax, and β-actin were purchased from Cell Signaling Technology (Beverly, MA, USA). The rabbit anti-goat, goat anti-mouse, and goat anti-rabbit secondary antibodies were purchased from Beyotime Institute of Biotechnology (Jiangsu, China).

### Experimental protocol

SD rats were randomly assigned into five groups including sham group, burn + vehicle group, burn + melatonin group, burn + melatonin + EX527 group and burn + EX527 group. Melatonin was first dissolved in ethanol to a concentration of 200 mg/ml, and then diluted in Ringer’s lactate solution yielding a stock concentration of 1 mg/ml. The sham group received the sham burns and no drug treatments; the burn + vehicle group were suffered from severe burns and treated with vehicle (1% alcohol in lactated Ringer solution); the burn + Mel group received the burns and melatonin; the burn + Mel + EX527 group received burns, melatonin and EX527 (5 μg, injected intraperitoneally) treatments; the burn + EX527 group received severe burn, 50 mg/kg body weight lactated Ringer solution and EX527 treatment. Drugs were administrated intraperitoneal at 0, 6 and 12 h following burns injury in burn + melatonin group and burn + melatonin + EX527 group.

### Histological examination and evaluation

Rats’ kidney specimens were fixed in 10% formalin, dehydrated in alcohol, embedded in paraffin, and then cut into 5-μm thickness sections and mounted. Sections were stained with hemotoxylin and eosin (H&E) after deparaffinization as described in other references^57^. Histological changes of glomerulus were scored based on the percentage of glomeruli necrosis, and these changes were ranked as 0: normal, 1: less than 25%, 2: 26–50%, 3: 50–75%, and 4: greater than 75%. The percentages of tubular containing casts were calculated as well. Ten high-magnification files for every slice were randomly selected for blinded observation.

### TUNEL staining for apoptosis

The commercial cell death detection kit was purchased from Roche Diagnostics (Indianapolis, IN, USA). The stained slices were observed and photographed under a microscope (All-in-one FSX100, Olympus, Japan). Three high-magnification files for every slice were randomly selected for blinded observation. The total cell numbers in each files were from 62 to 86. Every positive spot in DAPI staining was counted as a cell. The green spot overlapped with that was counted as apoptotic cell. The apoptotic index was determined as the percentage of apoptotic cells versus the total number of cells counted in a blinded manner.

### Renal function evaluation

Rat blood samples were collected to measure the serum levels of creatinine (Cr) and blood urea nitrogen (BUN) using a microplate reader (Infnite 200 PRO, Tecan, Switzerland).

### Lipid peroxidation and antioxidant enzymatic activity examination

Malondialdehyde (MDA), superoxide dismutase (SOD) and glutathione peroxidaseglutathione peroxidase (GSH) were determined to evaluate the oxidative status after stimulation. The renal tissue homogenate were reacted with a thiobarbituric acid reactive species (TBARS) assay kit (KeyGEN, Nanjing, China), and the reaction was used to obtain the tissue MDA, SOD and GSH levels (Nanjing, China) according to the manufacturer’s instructions. The absorbance values were measured using a microplate reader (Infnite 200 PRO, Tecan, Switzerland).

### Quantitative real-time PCR (qRT-PCR) analysis of renal tissue

The expression levels of TNF-α, IL-1β, IL-10, ICAM-1, Bcl-2, and Bax were analysed via qRT-PCR. Briefly, total RNA was isolated from tissues with TRIzol Reagent (Invitrogen, Carlsbad, CA, USA) and RNase-Free DNase I (Qiagen, Duesseldorf, Germany), and cDNAs were generated using SuperScript FirstStrand Synthesis System for RT-PCR (Invitrogen, Carlsbad, CA, USA). The concentrations and purities of RNA and cDNA were measured via BIO-RAD spectrophotometry (SmartSpecTM Plus, BIO-RAD, CA, USA). The primers (Table 1) were designed using Primer Premier 6.0 software and were synthesized by Shanghai Biological Engineering Co., Ltd. (Shanghai, China). PCR amplifications were conducted using the Power SYBR^®^Master Mix (Invitrogen, Carlsbad, CA, USA) in an iQ™5 Real-time PCR system (BIO-RAD, CA, USA). Relative quantification of the target gene expression levels was conducted using the 2^−∆∆Ct^ method.

### Western blotting analysis

To assess the protein levels of Bcl-2, cleaved-caspase-3, Ac-FoxO1, Ac-NF-κB, and Ac-p53, 50 μg of total protein were subjected to SDS-PAGE and transferred onto PVDF membranes. Membranes were blocked with 5% non-fat milk at room temperature for 3 h, incubated with primary antibodies specific to Bcl-2 (1:1000, Abcam, Cambridge, UK), cleaved-caspase-3 (1:1000, Abcam), Ac-FoxO1 (1:1000, Abcam), Ac-NF-κB (1:1000, Cell Signaling Technology, Beverly, MA), Ac-p53 (1:1000, Cell Signaling Technology), BAX (1:1000, Cell Signaling Technology), SIRT1 (1:1000, Cell Signaling Technology), or β-actin (1:1000, Abcam) at 4 °C overnight. Then, membranes were incubated with HRP-conjugated secondary antibodies diluted at 1:3000 (Boster, Wuhan, China) at 37 °C for 1 h. Protein bands on the membrane were visualized with ECL Kit (Millipore, USA) using FluorChem FC system (Alpha Innotech). Results were presented as densitometric ratio between the protein of interest and the loading control (β-actin).

### Statistical analysis

All data were presented as mean ± SEM. To evaluate the differences in the immunohistochemical staining, comparisons between two groups were conducted using Mann–Whitney U tests. Comparisons among multiple groups were assessed by one-way analysis of variance (ANOVA). Data were analyzed with the SPSS 13.0 program (IBM, Armonk, USA). *p* < 0.05 was accepted as statistically significant.

## Additional Information

**How to cite this article**: Bai, X.-Z. *et al*. Melatonin prevents acute kidney injury in severely burned rats via the activation of SIRT1. *Sci. Rep.* **6**, 32199; doi: 10.1038/srep32199 (2016).

## Supplementary Material

Supplementary Information

## Figures and Tables

**Table 1:** Primer sequences used for real-time-PCR analysis.

mRNA	Forward Primer	Reverse Primer:
SIRT1	5′-TCGTGGAGACATTTTTAATCAGG-3′	5′-GCTTCATGATGGCAAGTGG-3′
TNF-α	5′-TGCTTGTTCCTCAGCCTCTT-3′	5′-CAGAGGGCTGATTAGAGAGAGGT -3′
IL-1β	5′-GACCTGTTCTTTGAGGCTGAC-3′	5′-TCCATCTTCTTCTTTGGGTATTGTT-3′
IL-10	5′-CAGAGAAGCATGGCCCAGAA-3’	5′-GCTCCACTGCCTTGCTCTTA -3′
ICAM-1	5′-CACAAGGGCTGTCACTGTTCA-3′	5′-CCCTAGTCGGAAGATCGAAAGTC-3′
BAX	5′-GTGAGCGGCTGCTTGTCT-3′	5′-GGTCCCGAAGTAGGAGAGGA-3′
Bcl2	5′-GCGGA AGTCA CCGAA ATG-3′	5′-AGGAC AGCGA TGGGA AAA-3′
GAPDH	5′-GGCACAGTCAAGGCTGAGAATG-3′	5′-ATGGTGGTGAAGACGCCAGTA-3′
