# Supplementary material for: Melatonin prevents acute kidney injury in severely burned rats via the activation of SIRT1
Source: Sci Rep. 2016 Sep 7;6:32199. doi: 10.1038/srep32199 (PMC5013284; doi:10.1038/srep32199)
Supplement: Supplementary Information [file srep32199-s1.pdf]

Melatonin prevents acute kidney injury in severe burn rats via the activation of SIRT1

Xiao-Zhi Bai*, Ting He*, Jian-Xin Gao*, Yang Liu, Jia-Qi Liu, Shi-Chao Han, Yan Li, Ji-Hong Shi, Jun-Tao Han, Ke Tao, Song-Tao Xie, Hong-Tao Wang#, Da-Hai Hu#

Department of Burns and Cutaneous Surgery, Xijing Hospital, The Fourth Military Medical University, 127 Changle West Road, Xi'an, Shaanxi 710032, China

Short running title: Melatonin prevents kidney injury through SIRT1

*These authors contributed equally to this work.

#Corresponding authors: Da-hai Hu, Hong-Tao Wang

E-mail address: xjburnlab@163.com or burnlab@fmmu.edu.cn

Tel: +86-29-84775298

Fax: +86-29-83251734

Key Words: Melatonin, SIRT1, Kidney Injury, Burns, Inflammation, Oxidative Stress, Apoptosis

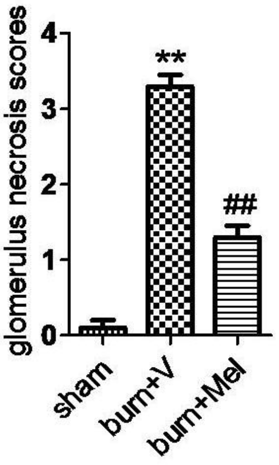

Figure S1 The glomerulus scores in each group. There were more necrotic glomerulus in burn + vehicle group than that in the burn + melatonin group (p<0.05). $^{**}p$< 0.05, compared to the value of sham group; $^{\#\#}p$< 0.05, compared to the value of burn + vehicle group.

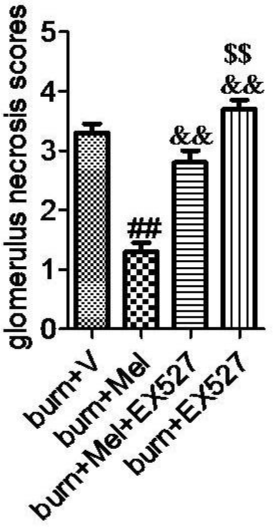

Figure S2 The glomerulus scores in each group. The administration of melatonin could reduce the necrosis of glomerulus while EX527 inhibited this effects even when EX527 was given to rats with melatonin (p<0.05). $^{\#\#}p$< 0.05, compared to the value of burn + vehicle group; $^{\&\&}p$< 0.05, compared to the value of burn + Melatonin group; $^{\$\$}p$< 0.05, compared to the value at burn + Melatonin + EX527 group.

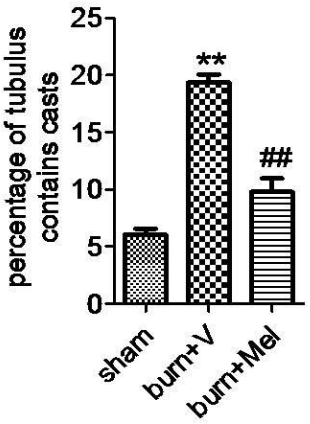

Figure S3 The percentage of tubulus containing casts in each group. There were more casts in burn + vehicle group than that in the burn + melatonin group (p<0.05). $^{**}p$< 0.05, compared to the value of sham group; $^{\#\#}p$< 0.05, compared to the value of burn + vehicle group.

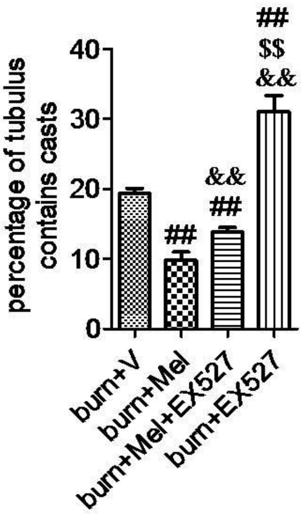

Figure S4 The percentage of tubulus containing casts in each group. The administration of melatonin could reduce the necrosis of glomerulus while EX527 inhibited this effects even when EX527 was given to rats with melatonin (p<0.05). $^{\#\#}p$< 0.05, compared to the value of burn + vehicle group; $^{\&\&}p$< 0.05, compared to the value of burn + Melatonin group; $^{\$\$}p$< 0.05, compared to the value at burn + Melatonin + EX527 group.

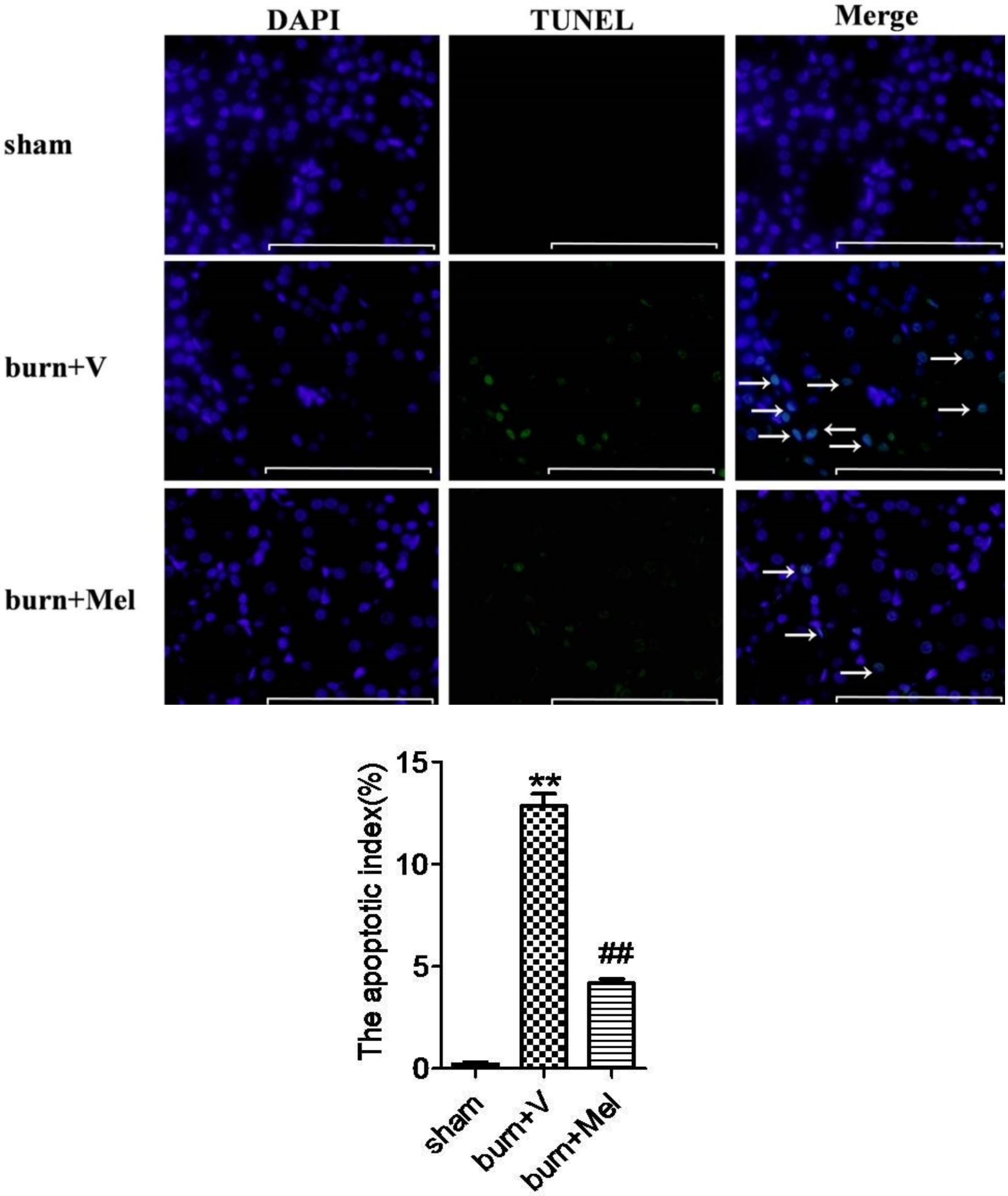

Figure S5 TUNEL assay for apoptosis. Twenty-four hours after the burn injury, rats were sacrificed and kidneys were harvested and subjected for TUNEL staining. TUNEL staining revealed increased numbers of apoptotic cells in burn group compared with sham group, while in burn + melatonin group, the apoptotic cells reduced significantly (p<0.05). White arrow: apoptotic cell. Scale bars in right lower corner represent 100μm. **p< 0.05, compared to the value of sham group; $^{\#\#}p$< 0.05, compared to the value of burn + vehicle group.
